# Role of B-Mode and Contrast-Enhanced Ultrasound in the Diagnostic Workflow of Gastro-Entero-Pancreatic Neuroendocrine Tumors (GEP-NETs)

**DOI:** 10.3390/cancers17111879

**Published:** 2025-06-04

**Authors:** Linda Galasso, Maria Grazia Maratta, Valeria Sardaro, Giorgio Esposto, Irene Mignini, Raffaele Borriello, Antonio Gasbarrini, Maria Elena Ainora, Giovanni Schinzari, Maria Assunta Zocco

**Affiliations:** 1 Department of Internal Medicine and Gastroenterology, Fondazione Policlinico Universitario Agostino, Gemelli IRCCS, Catholic University of Rome, 00168 Rome, Italy; linda.galasso@guest.policlinicogemelli.it (L.G.); giorgio.esposto@guest.policlinicogemelli.it (G.E.); irene.mignini@guest.policlinicogemelli.it (I.M.); raffaeleborr@gmail.com (R.B.); antonio.gasbarrini@unicatt.it (A.G.); mariaelena.ainora@policlinicogemelli.it (M.E.A.); 2 Medical Oncology Unit, Comprehensive Cancer Center, Fondazione Policlinico Universitario Agostino, Gemelli IRCCS, Catholic University of Rome, 00168 Rome, Italy; mariagrazia.maratta@guest.policlinicogemelli.it (M.G.M.); valeria.sardaro@guest.policlinicogemelli.it (V.S.); giovanni.schinzari@policlinicogemelli.it (G.S.); 3 CEMAD Digestive Disease Center, Fondazione Policlinico Universitario Agostino, Gemelli IRCCS, Catholic University of Rome, 00168 Rome, Italy

**Keywords:** gastro-entero-pancreatic neuroendocrine (GEP-NETS), contrast-enhanced ultrasound (CEUS), computed tomography (CT), magnetic resonance imaging (MRI)

## Abstract

Gastro-entero-pancreatic neuroendocrine tumors (GEP-NETs) represent a rare and varied class of neoplasms, characterized by diverse clinical presentations and prognostic trajectories. Accurate and prompt diagnosis is vital to inform and optimize therapeutic decisions. Ultrasound, including standard B-mode imaging and advanced methods such as contrast-enhanced ultrasound (CEUS) and endoscopic ultrasound (EUS), serves as a key component in the diagnostic evaluation of these tumors. B-mode US and CEUS provide non-invasive, accessible methods for early detection and characterization. On B-mode imaging, GEP-NETs typically present as well-defined, hyperechoic, or iso-echoic lesions, while CEUS highlights their characteristic vascularity, marked by arterial-phase hyperenhancement and venous-phase washout. Compared to CT and MRI, ultrasound offers real-time, dynamic imaging without ionizing radiation or nephrotoxic contrast agents, making it particularly advantageous for patients requiring frequent monitoring or with contraindications to other imaging modalities. CT and MRI are widely regarded as the preferred methods for staging and surgical planning due to their detailed anatomical visualization. However, ultrasound, especially CEUS, provides a significant adjunctive role in both early detection and the follow-up on GEP-NETs. This analysis delves into the strengths, challenges, and innovations in ultrasound technology for diagnosing pancreatic NETs, focusing on its contribution to comprehensive imaging strategies and its impact on patient care decisions.

## Simple Summary

Gastro-entero-pancreatic neuroendocrine tumors (GEP-NETs) are rare neoplasms requiring accurate diagnosis for optimal treatment. Ultrasound, including B-mode imaging, contrast-enhanced ultrasound (CEUS), and endoscopic ultrasound (EUS), aids in early detection and monitoring. While CT and MRI remain the gold standard for staging, ultrasound—especially CEUS—offers a radiation-free, real-time alternative. This review highlights ultrasound’s role in GEP-NET diagnosis and patient care.

## 1. Introduction

Neuroendocrine tumors (NETs) are a heterogeneous group of malignancies that can develop in various organs, most commonly in the gastrointestinal (GI) tract. NETs range from well-differentiated, slow-growing tumors to poorly differentiated, aggressive neuroendocrine carcinomas (NECs) [1,2]. They can be functional, producing hormones that cause distinct clinical syndromes (e.g., carcinoid syndrome), or non-functional, which often leads to delayed diagnosis [3].

Their rising prevalence and diagnosis at progressively younger ages have made them an emerging concern for clinicians in recent years [4,5]. The incidence of gastro-entero-pancreatic neuroendocrine tumors (GEP-NETs) has increased partly due to improved imaging techniques. According to the European Neuroendocrine Tumor Society (ENETS) guidelines, examination of the thorax, abdomen, and pelvis by contrast-enhanced computed tomography (CE CT scan) remains the gold standard imaging for diagnosis, staging, treatment response assessment, and surveillance [1]; however, other techniques can improve the aspects through the various phases of disease history. Indeed, a typical characteristic of GEP-NETs, especially the well-differentiated ones, is the expression of somatostatin receptors (SSTRs), which represent the ideal target for somatostatin receptor-based imaging techniques such as somatostatin receptor scintigraphy (SPECT) and the Positron emission tomography (PET) with 68 Ga-labeled somatostatin analogs used both for staging and therapeutic purposes. Thus far, SSTR-based PET/Tc is pivotal to select patients for cold or radiolabeled somatostatin analog treatments [6]. Another main feature of GEP-NETs is their extensive vascularization in both primary and metastatic lesions, especially liver metastasis [6].

Contrast-enhanced transabdominal ultrasound (CEUS) is gaining utmost importance in early detection and differential diagnosis of gastrointestinal masses. By the intravenous injection of microbubbles of hydrophobic gas encapsulated in a galactose, albumin or lipid shell, this imaging technique can provide information about organ perfusion and the micro-vascularization of liver and pancreatic lesions [7,8]. This approach offers a specific insight on tumor vascularization and it is an affordable, fast, and easily reproducible technique. Furthermore, the employed agent is not nephrotoxic, and patients are not exposed to ionizing radiation, as opposed to the CE CT scan.

Several studies have tried to establish the clinical value of CEUS in detection, differential diagnosis, assessment of treatment response, and even risk stratification of GEP-NETs. This review aims to provide a comprehensive overview of standard B-mode US and CEUS use in this setting based on the published literature and to offer practical insights into its current and future role in the diagnostic algorithm.

## 2. Ultrasound in the Evaluation of GEP-NETs

Transabdominal ultrasound (US) is often the first level of imaging for suspected gastrointestinal issues, including liver lesions. The overall sensitivity of US for detecting GEP-NETs ranges from 15 to 80% depending on tumor size and anatomical localization [9]. While the sensitivity of transabdominal US compared to other radiological imaging methods is relatively low, it still provides valuable insights into the morphological and vascular characteristics of NETs, especially when complemented by Color Doppler imaging [10,11].

Despite the fact that the specific ultrasound features of GEP-NETs may vary depending on their anatomical location, these tumors are typically hypoechoic and well-circumscribed, with an extensive vascularization, yet are influenced by their site within the gastrointestinal tract [12,13]. In Table 1, the B-mode ultrasound features of GEP-NETs are presented.

Transabdominal US effectiveness is further reduced in patients with excessive abdominal gas or in individuals with a large body habitus, as ultrasound beams undergo attenuation as they propagate through deeper tissues. Endoscopic ultrasound (EUS) is an evolution of conventional B-mode transabdominal US, integrating high-resolution ultrasonography with endoscopy to provide detailed, real-time imaging of the gastrointestinal wall and surrounding structures from within the lumen; this minimally invasive imaging modality allows us to obtain detailed images of the gastrointestinal tract and adjacent organs [14]. The use of a high-frequency EUS probe ranging from 5 to 10 MHz, and, in some cases, also high-frequency mini-probes (20–30 MHz), compared to 3–5 MHz conventional US probes, together with the close proximity of the transducer to the target lesion allows for an enhanced image resolution and a significantly greater sensitivity, making EUS particularly effective for identifying small lesions located in the pancreatic head or duodenal wall [9,15,16]. In recent years, EUS has evolved into an indispensable tool in the diagnosis and management of GEP-NETs, not only for its superior imaging capability but also for facilitating a range of ancillary techniques such as contrast-enhanced EUS (CE-EUS), EUS elastography, and EUS-guided fine needle aspiration (EUS-FNA) and fine needle biopsy (FNB). Among these, EUS-FNA and EUS-FNB have significantly enhanced the diagnostic yield, enabling cytological and histological evaluation with high sensitivity and specificity. EUS-FNB, in particular, provides a core tissue architecture that is crucial for accurate grading and Ki-67 index assessment and even molecular and immunohistochemical profiles that are relevant for guiding treatment planning [17,18]. CE-EUS and EUS elastography further improve lesion characterization by evaluating vascularity and stiffness, aiding differentiation from non-neuroendocrine lesions [19,20]. EUS is also pivotal in guiding therapeutic interventions, including ethanol ablation and radiofrequency ablation (RFA), particularly in patients unfit for surgery or with localized disease [21,22,23,24]. Overall, the integration of EUS with ancillary diagnostic and interventional techniques represents a pivotal advancement in the comprehensive management of GEP-NETs.

## 3. Comparison of CEUS and Traditional B-Mode US

Compared to traditional B-mode US, CEUS provides real-time, dynamic imaging of blood flow, enhancing the visualization of tumor perfusion patterns and improving lesion detection. A significant amount of research on contrast-enhanced ultrasound in neuroendocrine tumors primarily focuses on pancreatic neuroendocrine tumors (pNETs), benign or malignant liver lesions, and NET liver metastases.

### 3.1. CEUS in the Differential Diagnosis of pNETs and Other Pancreatic Lesions

Studies investigating pNETs using CEUS have consistently shown its sensitivity and specificity in differential diagnosis between pNETs and other pancreatic lesions, especially adenocarcinomas (PDACs). Yang D and colleagues described how a PDAC typically exhibits hypoenhancement during the arterial phase, often accompanied by necrotic areas, while a pNET usually presents with hyperenhancement or isoenhancement [25]. Another analysis conducted by Serra et al. in a cohort of 127 patients with solitary, undetermined pancreatic masses confirmed that CEUS enabled differentiation between exocrine and endocrine pancreatic tumor patterns, based on hypo- or hyperenhancement due to their lower or hypervascularity for PDACs and pNETs, respectively, with a sensitivity of 83.3% and a specificity of 60.0% [26]. Further, Malagò et al. investigated the correlation between B-mode ultrasound and CEUS features and tumor aggressiveness, indicated by higher Ki67% expression and the presence of liver metastases. B-mode US accurately identified only 3 out of 38 lesions as non-functional pNETs. In contrast, CEUS achieved a diagnostic accuracy of 63.1% by classifying lesions based on their enhancement pattern as hypervascular, isovascular, or hypovascular. Similarly, B-mode detected hepatic metastases in just 8% of cases, whereas CEUS identified them in 18.4%. Notably, all tumors confirmed as malignant and 12 out of 13 borderline lesions exhibited inhomogeneous enhancement on CEUS, demonstrating a significant correlation between enhancement patterns and Ki67% index expression. To note, all tumors with liver metastases displayed a hyperenhancing inhomogeneous pattern [27].

An important consideration in the evaluation of neuroendocrine tumors (NETs) is that contrast washout, typically regarded as a hallmark of malignancy, may also occur in benign lesions (benign pancreatic adenomas, chronic pancreatitis, or inflammatory masses) or well-differentiated NETs. Washout refers to the process by which a lesion loses contrast enhancement over time after the initial arterial phase uptake. In CEUS, this typically means that the lesion initially appears hyperenhanced (brighter) compared to surrounding tissue but then becomes iso- or hypoenhanced (darker) during the portal or late phases as the contrast agent clears from the lesion faster than from the adjacent normal tissue.

Washout is a multifactorial, dynamic process reflecting tumor vascular architecture, blood flow, and tissue characteristics. This phenomenon can be attributed to the intrinsic hypervascularity of these tumors, which possess a well-preserved capillary network that allows for rapid contrast influx and efflux. In CEUS using agents such as SonoVue, which remain confined to the intravascular space, washout patterns are entirely perfusion-dependent rather than influenced by interstitial diffusion [28,29]. While malignant lesions are often characterized by early and marked washout, benign or well-differentiated NETs can similarly exhibit washout, albeit with distinct features. Typically, these lesions show rapid, homogeneous enhancement in the arterial phase, followed by mild and delayed washout, usually occurring between 60 and 960 s. This reflects their intact microvascular structure and lack of significant fibrotic stroma. In contrast, poorly differentiated or malignant NETs frequently demonstrate heterogeneous arterial enhancement and early, intense washout, corresponding to necrosis, aberrant angiogenesis, and a disrupted stromal architecture [28].

Therefore, the contrast-enhanced ultrasound (CEUS) provides valuable insights into the vascular characteristics of pancreatic neuroendocrine tumors (pNETs), helping to distinguish between low-to-intermediate grade (G1/G2) tumors and high-grade carcinomas (G3/pNETCs). G1 and G2 pNETs typically appear as well-defined, hyperenhancing lesions in the arterial phase due to their rich capillary networks and often show homogeneous enhancement with slow washout in the late phase. In contrast, G3 pNETCs, which are poorly differentiated and more aggressive, frequently demonstrate hypoenhancement or heterogeneous enhancement in the arterial phase, reflecting reduced vascularity and the presence of necrotic or cystic areas. These high-grade tumors also tend to wash out contrast rapidly, becoming hypoenhancing in the portal or late phase [30].

**Figure 1:**
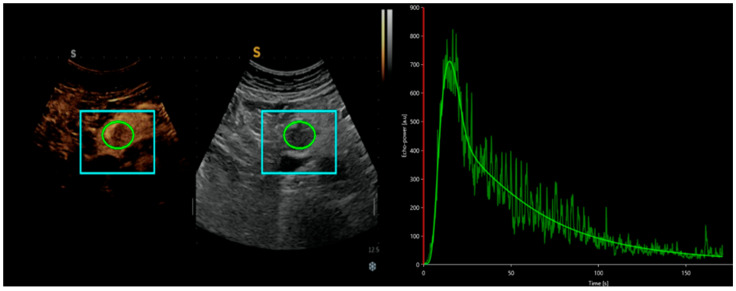
Representation of an analysis of a CEUS clip of a pancreatic lesion with Vuebox software, from which a time-intensity curve is obtained, providing numerical values such as peak enhancement, area under the curve, and mean contrast agent transit time.

In addition to playing a role in qualitative differential diagnosis, CEUS also enables a dynamic quantitative analysis of the US clip. Using Vuebox software, intensity-time curves (TICs) can be obtained, providing numerical, quantitative, and objective data on the contrast agent behavior in a specific lesion [Figure 1].

In this context, the group led by Yang DH et al. demonstrated the use of D-CEUS in analyzing the relationship between the US clip and the grading of NETs. In their population of 42 patients undergoing surgery, they observed that the majority of G1/G2 pNETs showed higher TICs than pancreatic parenchyma, whereas G3/pNECs had lower TICs. By converting these data into quantitative values, they found that CEUS parameters, such as peak enhancement (PE), area under the curve (AUC), and mean transit time (mTT), were significantly higher in G1/G2 pNETs compared to G3/pNECs, with PE and AUC offering high sensitivity and accuracy for predicting G3/pNECs [31].

In Table 2 are listed the ultrasound behaviors of GEPNETs after the administration of the contrast agent.

### 3.2. CEUS in the Differential Diagnosis of NETs, Liver Metastases, and Other Liver Lesions

As previously mentioned, CEUS plays a crucial role in the differential diagnosis between hepatic metastases from pancreatic NETs and benign or malignant hepatic lesions.

As demonstrated in the study by Massironi S. et al., these metastases exhibit a different behavior compared to those originating from other primary tumors, showing an increased arterial enhancement on CEUS, a pattern similar to that of a hepatocellular carcinoma and opposite to that of other solid tumor metastases [32] [Table 2].

It is also possible to perform a differential diagnosis between primary liver malignancies such as HCC and intrahepatic cholangiocarcinoma (iCCA) and liver metastases from neuroendocrine tumors (NETs), based on their distinct enhancement patterns.

HCC typically presents an homogeneous hyperenhancement during the arterial phase, followed by a distinct washout in the portal and late phases, features that are characteristic of primary liver cancer [33]. In contrast, NET metastases commonly display a peripheral, nodular, and discontinuous enhancement in the arterial phase, with a gradual centripetal fill-in [29]. Notably, unlike HCC, NET metastases could lack a washout in the later phases, reflecting their slow-flow vascular profile and supporting accurate lesion differentiation [34]. Similarly, CEUS allows for differentiation between NET metastases and iCCA. iCCA is typically characterized by rim-like arterial phase enhancement with early and pronounced washout in the portal and late phases, features that are associated with its hypovascular, fibrotic composition. These patterns contrast with the persistent, centripetal enhancement and absence of washout seen in NET metastases [35].

In the case of differentiating NET liver metastases from benign liver lesions such as hemangiomas or adenomas, CEUS captures distinct vascular signatures that serve as key criteria for non-invasive differentiation between these conditions [36].

Hepatic hemangiomas typically display a different enhancement pattern, characterized by peripheral, nodular, and discontinuous enhancement during the arterial phase, followed by progressive centripetal fill-in over time, while showing persistent or even increased enhancement, reflecting their slow-flow vascular nature [37].

Hepatic adenomas, although generally hyperenhancing in the arterial phase, tend to show little or no washout and remain isoenhancing or slightly hypoenhancing in the later phases. This absence of washout is a vital distinguishing feature, indicating the benign nature of adenomas. Among adenoma subtypes, inflammatory adenomas may exhibit persistent enhancement, occasionally resembling focal nodular hyperplasia (FNH), but they do not demonstrate the mild washout seen in NET metastases [38].

Focal nodular hyperplasia (FNH) presents a distinct enhancement pattern on CEUS, with rapid, homogeneous hyperenhancement during the arterial phase and a characteristic ‘spoke-wheel’ appearance due to the central fibrous scar. FNH lesions generally maintain their enhancement in the portal and late phases, often appearing iso- or slightly hyperenhancing compared to the surrounding liver tissue, without the washout observed in NET metastases. The consistent enhancement throughout all phases, combined with the central scar, enables clear differentiation from NET metastases, particularly when imaging features are closely analyzed [39].

In Table 3, the CEUS features of the main liver lesions are presented.

### 3.3. CEUS Characteristics of NETs Associated with Medical Therapies

Since the antiproliferative activity of somatostatin analogs has been extensively studied in numerous phase two studies, and more recently in two randomized phase three trials, the PROMID (placebo-controlled, prospective, randomized study in patients with metastatic neuroendocrine midgut tumors) and the CLARINET (controlled study of lanreotide antiproliferative response in neuroendocrine tumors) [40,41,42], the group led by M. del Prete conducted a retrospective study evaluating whether the modifications in the microvascularization of NET lesions could be a predictor of response to treatment, performing CEUS at the diagnosis and at 3, 6, and 12 months after the start of somatostatin analog (SSA) therapy. Their population included 13 patients with only pancreatic lesions, 8 patients with only hepatic lesions and 26 with both, with a total of 99 lesions evaluated. At baseline, all lesions showed a hypervascular pattern: 39 (90.7%) of pancreatic lesions appeared to be homogeneous and 4 (9.3%) appeared to be inhomogeneous, while 47 (83.9%) of hepatic lesions appeared to be homogeneous and 9 (16.1%) appeared to be inhomogeneous. At baseline, they found a significant association between a CEUS hypervascular homogeneous pattern and the absence of necrosis at CT scan (x^2^ = 79.0: *p* < 0.0001) and with Ki67 value (x^2^ = 24.5, *p* < 0.0001), with a homogeneous hypervascular pattern correlating with a low tumor grade (x^2^ = 24.0, *p* < 0.0001).Particularly interesting are the data concerning the response to SSA therapy: the appearance of an inhomogeneous hypervascular pattern was observable in 10 pancreatic lesions and 10 liver lesions at 3 months after the start of therapy, in 22 liver lesions and 18 pancreatic lesions at 6 months, and in 24 liver lesions and 20 pancreatic lesions at 12 months, respectively.

A statistically significant association was found between the appearance of an inhomogeneous hypervascular pattern in CEUS at 6 and 12 months after the start of SSA therapy and tumor necrosis visible on CT, considered as disease response to therapy. Moreover, by comparing radiological findings on CEUS at 3 months and tumor response in CT at delayed timepoints (6 and 12 months), a statistically significant correlation was demonstrated between an US inhomogeneous pattern and disease response visible on a CT scan. These results confirm the hypothesis that CEUS might be not only a valid predictor of tumor aggressiveness, but also an early predictor of response to treatment compared to a CT scan [30].

Another study investigated whether the change in lesion perfusion shown on CEUS could be a predictive marker of response even in patients treated with PRRT. Before therapy, liver metastasis showed the typical hyperenhancing pattern during the arterial phase of CEUS. A decrease in tumor vascularity was visible on both CT and CEUS six weeks after treatment and was considered a sign of response to PRRT. This is particularly relevant if we consider that morphological changes in response to therapy are visible on CT at least six months after therapy [43]. CEUS could offer high temporal resolution and dynamic assessment of microvascularization patterns in real time, allowing for precise tumor perfusion characterization and therapy response even after local ablative treatment or novel anti-angiogenetic therapies, as demonstrated by the experience in other gastrointestinal cancers [44,45,46].

These findings highlight the growing role of CEUS not only in the differential diagnosis of neuroendocrine tumors but also in treatment monitoring and follow-up, offering a non-invasive, real-time imaging tool that enhances clinical decision-making.

Eventually, CEUS may find an intra-operative application for the detection and characterization of tumor lesions in many gastrointestinal solid-tumors: the intra-operative use of high-resolution linear transducer techniques with CEUS can detect additional small tumor lesions (diameter < 1 cm) not previously clearly diagnosed during the pre-operative work-up and then histologically confirmed by intra-operative biopsy or after surgical resection [47]. This application is of paramount importance giving the importance of liver cytoreduction in GEP-NETs, especially in functionating a tumor for symptom control [1].

## 4. Comparison of Advanced Imaging Techniques: Evaluating the Role of CEUS Versus CE CT Scan, MRI, and PET/CT

Despite the fact that comprehensive radiological and nuclear medical diagnostic imaging play an important role in the standard work-up of patients with GEP-NETs, the sonographic examination, including CEUS, is increasingly recognized as a valuable imaging tool to screen and follow-up for these neoplasms, especially for liver metastases.

The sensitivity for detecting liver metastasis has been reported to be comparable to multiphasic CT scan [30]. Its ability to differentiate hypervascular liver metastases from benign hepatic lesions makes it a compelling alternative, particularly in patients with contraindications to iodinated or gadolinium-based contrast agents and it is easily repeatable at different timepoints of disease history without concerns about side effects (cumulative radiations, contrast medium allergy, renal impairment, and others), aside from the anecdotical incidence of a severe hypersensitivity reaction (reported in about 0.002% in large-scale studies [48,49]. However, CEUS is inherently operator-dependent, has a limited field of view, and is less effective for deep-seated or extrahepatic tumors, reducing its utility for comprehensive staging at baseline. Moreover, no unanimous strategy exists concerning the most appropriate timing schedule for performing CEUS to assess therapy response or even as an alternative follow-up strategy to spare CT or MRI radiation to the patients. In fact, the studies examined used heterogeneous disease timepoints specifically designed to answer their own clinical question and no recommendation or guideline are available. Yet, CE CT and magnetic resonance imaging (MRI) remain the gold standards for the initial diagnosis and staging of GEP-NETs [1], with MRI showing superior sensitivity compared to CT in detecting small liver metastases due to its higher contrast resolution, especially with diffusion-weighted imaging (DWI) [50,51,52]. CT, on the other hand, is more widely available, cost-effective, and preferred for whole body evaluation, particularly for detecting lymph node involvement and distant metastases. Nevertheless, both modalities have limitations in identifying small, occult, or functionally active NET lesions, which is where somatostatin receptor (SSTR)-based imaging, such as positron emission tomography (PET) with Gallium-68 (68Ga) or Copper-64 (64Cu) DOTATATE, plays a crucial role. Indeed, SSTR PET/CT (or PET/MRI) is the most sensitive and specific technique for NETs, particularly for well-differentiated, SSTR-expressing tumors [53]. It provides superior lesion detection compared to CT or MRI, especially in the setting of metastatic disease, where it significantly alters treatment strategies. The main advantages of PET include its ability to detect occult lesions, quantify tumor burden, and guide peptide receptor radionuclide therapy (PRRT) eligibility [54]. Still, SSTR PET may lack uptake in poorly differentiated or high-grade lesions. In the latter scenario, 18F Fluorodeoxyglucose (18F-FDG) PET is complementary in that it detects aggressive, poorly differentiated disease with a higher grade and worse prognosis [55]. Dual functional imaging allows for the non-invasive characterization of the functional status and tumor heterogeneity based on the analysis of the uptake intensity of target-specific radiotracers [56,57]. However, its high cost, limited availability, and reliance on specialized radiotracer production facilities restrict its widespread use. Also, to date, the role of SSTR PET in response assessment and predicting outcome remains under evaluation [58].

From a cost-effectiveness perspective, CEUS remains advantageous as a first-line or follow-up modality in liver-dominant disease due to its affordability [59], absence of ionizing radiation, and repeatability. In contrast, CE CT and MRI provide broader anatomical assessment and are indispensable for staging, while PET is reserved for cases requiring high diagnostic certainty, particularly when conventional imaging is inconclusive or for therapy planning. The optimal imaging strategy depends on the clinical context, tumor grade, metastatic burden, and resource availability, often requiring a multimodal approach in which CEUS may have a crucial role to maximize diagnostic accuracy and guide appropriate management.

## 5. Conclusions

Over the years, studies confirmed the reliability of CEUS in initial tumor diagnosis, distinguishing metastatic lesions, and assessing treatment response by evaluating disease vascular pattern changes, complete tumor necrosis, and residual viable tissue. CEUS served a safer alternative to CE radiological exams for monitoring therapy outcomes even earlier and immediately post-treatment administration. Advanced CEUS techniques, including D-CEUS and TIC computed analysis, enhance tumor visualization and risk stratification. These innovations aid in optimizing individualized treatment, minimizing unnecessary toxicity and costs. While CEUS holds great promise for clinical practice, further research is needed to further refine its role in GEP-NET management and provide clear recommendations on therapy follow-up.

## Figures and Tables

**Table 1:** B-mode ultrasound characteristics of GEP-NETs according to anatomical site of origin.

Location	Margins	Echogenicity	Echotexture	Color Doppler
Stomach (Gastric NETs)	Well-defined, sometimes polypoid	Hypoechoic compared to the gastric wall	Homogeneous in well-differentiated NETs, heterogeneous in high-grade NETs	Moderate to high vascularization
Small Intestine (Enteric NETs, si-NET)	Well-defined, sometimes with a peritumoral hypoechoic halo	Hypoechoic compared to the intestinal wall	Homogeneous in low-grade NETs, heterogeneous in aggressive NETs	Marked intralesional vascularization
Colon-Rectum (Colorectal NETs)	Well-circumscribed, often small (<1 cm)	Hypoechoic or isoechoic	Homogeneous in low-grade NETs, heterogeneous in poorly differentiated NETs	Moderate vascularization
Pancreas (Pancreatic NETs, pNETs)	Well-defined, sometimes lobulated	Hypoechoic compared to pancreatic parenchyma	Homogeneous in well-differentiated forms, heterogeneous in high-grade NETs	Increased intralesional vascularization, often intense

**Table 2:** The CEUS characteristics of GEP-NETs.

Type of Lesion	Arterial Phase	Portal/Late Phase	Additional Considerations
Pancreatic Adenocarcinoma	Hypoenhancement (reduced contrast)	Persistent hypoenhancement	Often associated with necrotic areas
Pancreatic Neuroendocrine Tumor (pNET) G1/G2	Hyperenhancement or Isoenhancement	May retain a higher signal than pancreatic parenchyma	Hypervascular lesion; higher TIC parameters (PE, AUC, mTT)
Pancreatic Neuroendocrine Tumor (pNET) G3	Hypoenhancement	Persistent hypoenhancement	Lower TIC parameters (rPE, rAUC) compared to G1/G2 pNETs
Pancreatic Neuroendocrine Carcinomas (pNEC)	Hypoenhancement	Persistent hypoenhancement	Lower TIC parameters (rPE, rAUC) compared to G1/G2 pNETs
Liver metastases from gastrointestinal NETs	Increased arterial enhancement	Persistent hypervascularization	Pattern similar to HCC, opposite to other liver metastases

**Table 3:** CEUS features of hepatocellular carcinoma, cholangiocarcinoma, and metastases from gastrointestinal and neuroendocrine tumors.

Lesion Type	Arterial Phase	Portal Phase	Late Phase	Typical Appearance
Hepatic Hemangioma	Peripheral nodular enhancement (discontinuous)	Progressive centripetal fill-in	Complete fill-in (isodense with liver)	Well-defined, slow filling from periphery to center
Hepatic Adenoma	Hyperenhancing (often heterogeneous)	Becomes iso- or hypoattenuating	No washout (hypodense)	Hypervascular lesion with early enhancement and washout
Focal Nodular Hyperplasia (FNH)	Homogeneous intense enhancement, central scar hypodense	Isoattenuating	Central scar enhances (hyperintense)	Spoke-wheel arterial pattern, central scar visible in delayed phase
Hepatocellular Carcinoma (HCC)	Arterial hyperenhancement (early, intense)	Washout (hypodense relative to liver)	Late and mild washout	Classic pattern: arterial hyperenhancement + portal/late phase washout
Intrahepatic Cholangiocarcinoma (iCC)	Mild peripheral enhancement	Progressive centripetal enhancement	Marked washout	Peripheral rim enhancement with delayed fibrous core uptake
